# Studying effect of respiration on total cavopulmonary connection flows using real-time cardiac magnetic resonance

**DOI:** 10.1186/1532-429X-16-S1-P137

**Published:** 2014-01-16

**Authors:** Reza H Khiabani, Kevin K Whitehead, Jefferson D Losse, Mark A Fogel, Ajit Yoganathan

**Affiliations:** 1 Department of Biomedical Engineering, Georgia Institute of Technology & Emory University, Atlanta, Georgia, USA; 2 Division of Cardiology and Department of Radiology, Children's Hospital of Philadelphia, Philadelphia, Pennsylvania, USA

## Background

Accurate measurement of vessel flows in the Total Cavopulmonary Connection (TCPC) can lead to better estimation of hemodynamics, which has been related to clinical outcomes in single ventricle patients. CMR is usually acquired in breath held conditions; however, respiratory effects can be considerable since the blood is passively routed to the lungs in Fontan patients. Here, we assessed the effect of respiration on caval and aortic flows using real time CMR images.

## Methods

**Figure 1:**
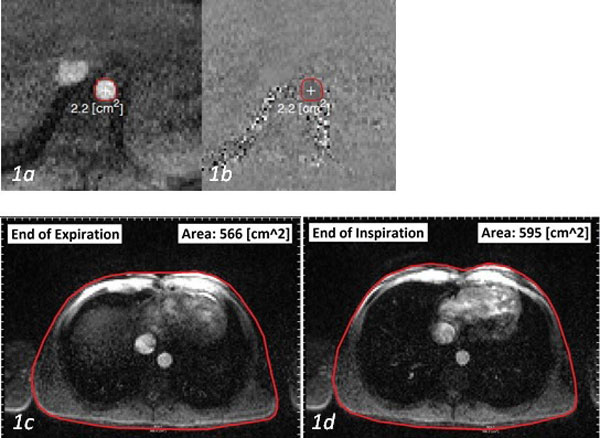
**Positioning of the region of interest when mapping IVC flow rate (a, b); and sample chest wall motion tracking at the end of expiration (c), and the end of inspiration (d) showing the minimum and maximum areas of the thoracic cavity during respiration, respectively**.

Eight single ventricle patients with TCPC palliation underwent resting cardiac magnetic resonance imaging at both free breathing (FB) and breath held (BH) conditions. Real time, through-plane phase encoded velocity mapping (PC-MRI) was performed in superior (SVC), and inferior (IVC) vena cava, and ascending (AAO) and descending (DAO) aorta. FB and BH resting flow (Q) waveforms were then obtained at each vessel (Figure 1-a, b). Respiratory cycle, inspiration and expiration periods were determined by tracking chest wall motion from the PC-MRI magnitude images (Figure 1-c, d). Pulsatility Index was calculated as PI=(Q_max-Q_min)/(2×Q_mean)×100 for a Cardiac (PI_card) or Respiratory (PI_resp) cycle. Significance of the difference in cycle-averaged vessel flows and PI between FB and BH conditions were investigated using paired t-test (*p < 0.05).

## Results

**Figure 2:**
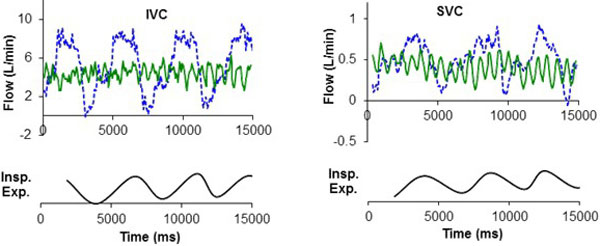
**Sample resting flow waveforms of IVC (top-left) and SVC (top-right) at free breathing (dashed line) and breath held (solid line) conditions, along with the respiratory cycle determined by tracking the chest wall motion (bottom)**.

Mean flows in all the vessels trended toward an increase with respiration compared to the BH conditions; however, only the increase in SVC flow was significant (1.5 vs. 1.3 L/min, *p < 0.05). IVC and SVC flows were markedly phasic with the respiratory cycle (Figure 2), and their flow pulsatility increased with respiration (PI_resp/PI_card = 1.0 ± 0.1, 0.9 ± 0.2, *2.0 ± 0.7, and 1.5 ± 0.1 in DAO, AAO, IVC, and SVC, respectively). The IVC and SVC flows were significantly higher at inspiration compared to expiration (*p < 0.05). On the other hand, no significant effect of respiration was observed on the AAO and DAO flow waveforms (Q_insp/Q_exp at FB [BH] = 1.0 ± 0.0[1.0 ± 0.1], 1.1 ± 0.1[1.0 ± 0.1], *1.8 ± 0.5[1.0 ± 0.1], and *1.7 ± 0.3[1.0 ± 0.1] in DAO, AAO, IVC, and SVC, respectively).

## Conclusions

A novel technique of measuring flow and tracking respiration with the same slice plane was presented, obviating the need for respiratory bellows. Using this technique, we demonstrated that respiratory effects dominate the venous flow but have little effect on systemic arterial flow in Fontan patients. However, the effect of breath holding on mean flows is fairly small, supporting the routine use of breath holding in assessing flows in single ventricle patients.

## Funding

This study was supported by the National Heart, Lung, and Blood Institute Grants HL67622, HL098252, and HL089647.

